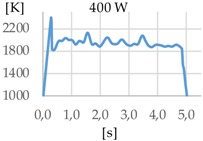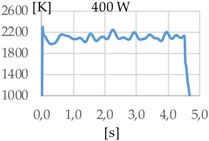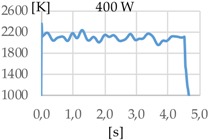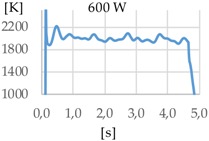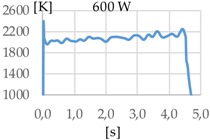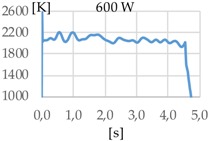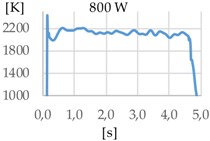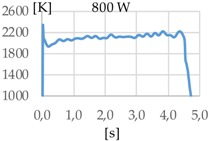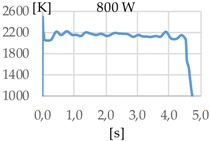# Study of the Influence of Shielding Gases on Laser Metal Deposition of Inconel 718 Superalloy

**DOI:** 10.3390/ma11081388

**Published:** 2018-08-09

**Authors:** Jose Exequiel Ruiz, Magdalena Cortina, Jon Iñaki Arrizubieta, Aitzol Lamikiz

**Affiliations:** Department of Mechanical Engineering, University of the Basque Country, Plaza Torres Quevedo 1, 48013 Bilbao, Spain; magdalena.cortina@ehu.eus (M.C.); joninaki.arrizubieta@ehu.eus (J.I.A.); aitzol.lamikiz@ehu.eus (A.L.)

**Keywords:** LMD, laser metal deposition, shielding gas, argon, helium, additive manufacturing, Inconel 718, melt pool temperature

## Abstract

The use of the Laser Metal Deposition (LMD) technology as a manufacturing and repairing technique in industrial sectors like the die and mold and aerospace is increasing within the last decades. Research carried out in the field of LMD process situates argon as the most usual inert gas, followed by nitrogen. Some leading companies have started to use helium and argon as carrier and shielding gas, respectively. There is therefore a pressing need to know how the use of different gases may affect the LMD process due there being a lack of knowledge with regard to gas mixtures. The aim of the present work is to evaluate the influence of a mixture of argon and helium on the LMD process by analyzing single tracks of deposited material. For this purpose, special attention is paid to the melt pool temperature, as well as to the characterization of the deposited clads. The increment of helium concentration in the gases of the LMD processes based on argon will have three effects. The first one is a slight reduction of the height of the clads. Second, an increase of the temperature of the melt pool. Last, smaller wet angles are obtained for higher helium concentrations.

## 1. Introduction

Laser Metal Deposition (LMD) is an Additive Manufacturing (AM) technology that consists on the deposition of material layers that are melted by a laser source. This process belongs to the Directed Energy Deposition (DED) technology group and enables working with a very wide range of metal materials. The filler material to be deposited is usually supplied in the form of powder and is conducted through a nozzle into the melt pool, which has been created on a substrate surface by a laser beam. In this process, the powder is melted and deposited by creating a new layer of material. Subsequent layers generate geometries, enabling the addition of features to existing parts or the manufacturing of new ones [1].

The entire process is carried out employing two different gas flows: a shielding gas, whose function is the generation of a protective atmosphere so that oxidation reactions are avoided, and a carrier gas that is used to transport the powder through the entire circuit and nozzle to the melt pool. The use of these two different gas flows has direct consequences on the process. Firstly, similar to other welding processes, the shielding gas is necessary to protect the deposited material from oxidation [2]. It represents the largest gas flow used and it has a direct impact on the quality of the deposited material, mainly in the porosity generation, but this phenomenon can be easily avoided by parameters modification [3,4]. Secondly, the carrier gas drags the powder from the powder feeder to the nozzle and into the melt pool. This gas flow accelerates and injects the powder particles at the nozzle exit. The interaction of both, carrier and shielding gas, with the powder and the atmosphere determines the powder distribution in the focal point of the nozzle. This powder distribution is a key factor for the LMD process efficiency [5,6]. Bibliography of LMD process situates argon as the most common gas (both for protective and drag gas), followed by nitrogen. This is due to the fact that nitrogen is mostly unreactive and more economical than argon, but does not always protect the process from chemical reactions, since nitrogen reacts with Ti, Nb, and V [7].

The use of the LMD technology as a manufacturing and repairing technique in industrial fields, like the die and mold and aerospace industry, is increasing within the last years. In these industrial sectors, the process is specially focused on different steel alloys, nickel superalloys [8,9] and titanium alloys. Steels are usually alloyed with vanadium, nickel superalloys, like Inconel 718 or Inconel 625, have titanium and niobium in its chemical composition and one of most typical titanium alloys is Ti6Al4V, which contains both titanium and vanadium. Thus, in these cases nitrogen should not be used as shielding or dragging gas on LMD process.

There are many similarities between the LMD process and Laser Beam Welding in terms of shielding gases. In fact, both use the same heat source and materials must be protected from chemical reactions with a shielding gas. Different research work carried out in the field of Laser Beam Welding states that the use of argon with high densities of energy can lead to plasma formation due to the lower ionization energy of argon compared to other gases, like helium [10]. This plasma formation creates a shield that blocks the laser beam [11], thus reducing the energy that finally reaches the workpiece. On the one hand, the depth of the welds using an argon-helium mixture is comparable to those that are achieved with a pure argon flow. On the other hand, the width is bigger when the argon-helium mixture is used [10].

As previously mentioned, argon and not nitrogen is usually employed for LMD. In fact, machine tool builders working with LMD process, like DMG MORI (DMG MORI, Bielefeld, Germany), Trumpf (TRUMPF GmbH + Co., KG, Ditzingen, Germany), or MAZAK (Yamazaki Mazak Corporation, Oguchi, Japan), among others, usually recommend the use of argon despite its high price in order to avoid the risk of unwanted reactions in the process. However, some of these leading companies have started to use helium and argon as carrier and shielding gas, respectively. This is due to a fluid dynamical issue: helium’s density is lower than argon’s, so when the helium flow crosses the argon one, turbulences are reduced, and a more stable gas flow is achieved at the substrate. This phenomenon helps to improve the powder concentration and protective gas concentration at the melt-pool, hence simplifying the nozzle design.

Therefore, there is a necessity to know how the use of different gases may affect the LMD process. From the literature review, it is noted that some research has been realized focusing on the use of argon or nitrogen individually, with the argon case the most documented. However, there is a lack of information about the influence that helium or gas mixtures may have on the LMD process.

The aim of the present work is to evaluate the effect of a mixture of argon and helium on the LMD process by analyzing single tracks of Ni based alloy Inconel 718. The height, width, and depth of the clads, along with the temperature of the process, are measured for the different gas mixtures employed.

## 2. Materials and Methods

The selected material for this work is a nickel-based superalloy, Inconel 718. This material is commonly used in the aerospace industry for turbine and other structures where temperatures can be higher than 600 °C. It is probably one of the most typical materials used in LMD process. Thus, substrate material is an Inconel 718 alloy and powder material is an Oerlikon MetcoClad 718 (Oerlikon, Freienbach, Switzerland), which has been used as the filler material. Both share the same chemical composition within certain compositional limits, as shown in Table 1 and Table 2. Powder particle size distribution is shown in Table 3, in order to enable other fellow researchers to replicate the same tests.

All of the tests that are described within this work were carried out on a laser processing cell, Kondia Aktinos 500 (Kondia, Elgoibar, Spain), rebuilt from a conventional milling center. It has three linear plus two rotary axes for a total of five-axis cinematics, and a work volume of 700 × 360 × 380 mm^3^. The laser source is a Rofin FL010 (ROFIN-SINAR, Bergkirchen, Germany), a Yb:YAG fiber laser of 1 kW of maximum power and 1070 nm wavelength. An optical multi-mode fiber is used to guide the laser beam to the processing cell and an optical lens focuses it at 200 mm, thus creating a circular spot of 1.6 mm of diameter, approximately. In addition, the powder feeder employed is a Sulzer Metco Twin 10-C (Oerlikon Metco, Pfäffikon, Switzerland), which can be used with argon, nitrogen, and other gases, like helium. The powder is injected by means of a self-developed coaxial nozzle EHU/Coax 2015 (UPV/EHU, Bilbao, Spain) [5].

The gases that were used during the experimental tests, supplied by Praxair (Praxair, Inc., Danbury, USA), are (1) Argon, with a purity of 99.998%, (2) Helistar 25, whose composition is argon 75% and helium 25%, and (3) Helistar 50, which is a half argon and half helium mixture [14,15,16]. The same gases were supplied both as shielding and as carrier gas, in order to avoid mixing and knowing the precise composition.

Two different experiments were designed. One was intended to measure the melt pool temperature and its cooling time. The second one aimed to characterize the deposited tracks by measuring their height, width, dilution depth, and wet angle, along with the temperature of the process. The temperature was measured by means of a digital two-color pyrometer with an IGAR 12-LO (LumaSense Technologies, Inc., Santa Clara, CA, USA) optic fiber [17], which was focused on the same point as the laser beam. The use of this kind of technique instead of a standard one-color pyrometer is because the measurement of a two-color pyrometer is independent of the emissivity in a wide range of temperature and it is unaffected by fume or powder in this case.

### 2.1. Melt Pool Temperature Measurement and Cooling Time

The tests were realized on a 10 mm thickness substrate in order to avoid considerable thermal affection. Previously, the test specimens were cleaned and prepared so that a homogeneous surface was attained. The k-factor (also known as emissivity slope) of the two-color pyrometer was calibrated with a similar substrate of equal dimensions and characteristics heated in a furnace (Helmut ROHDE GmbH, Prutting, Germany) up to 1423 K and then measured with a thermocouple type K. The laser was set at the focal distance of the nozzle with the dragging and shielding gas flows on, while a laser power of 250 W was used to heat the surface for 1 s, so that the melting point was reached.

**Figure 1:**
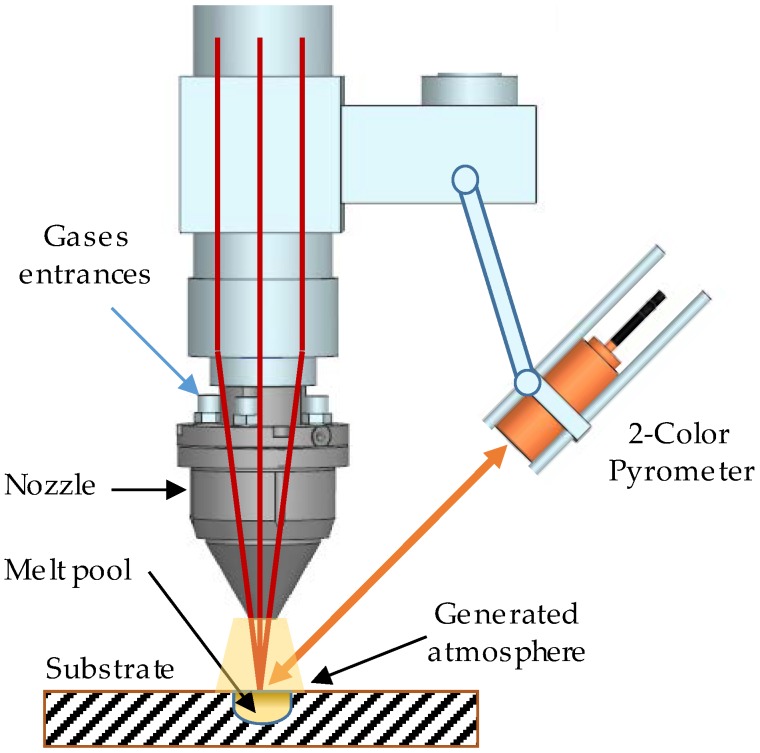
Experimental setup.

Once the laser power was off, the measurement of the temperature continued until the pyrometer stopped registering any signal intensity from the substrate. The time between measurements was set to 16 ms, as it showed stability and compromise with immediate measurements. The three different gases (Argon, Helistar 25 and Helistar 50) were tested with four repetitions of the measurement for each of them. The Figure 1 shows the experimental setup.

### 2.2. Laser Metal Deposition Experiments

The same substrate preparation for these experiments were carried out. For the data record and subsequent analysis, three tracks were deposited with pure argon, so that they could be used as the reference tracks. The process parameters were also set with reference values, in order to test the different gases with the same conditions. The parameters were selected from previous works in order to avoid cracks and porosity, and they are shown in Table 4.

**Figure 2:**
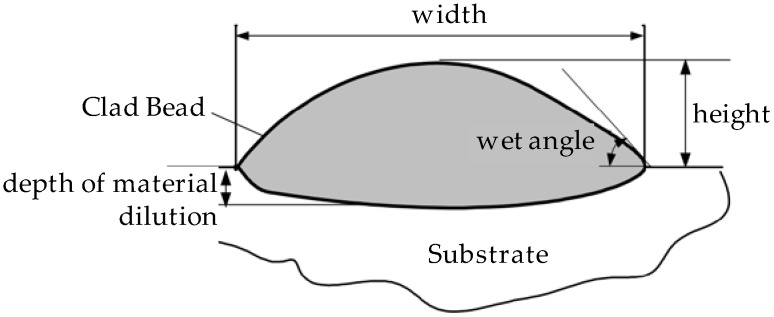
Clad cross section parameters.

Once the reference tests were carried out, the same process parameters were used for testing the two gas mixtures, Helistar 25 and Helistar 50, with 25% and 50% of helium concentration, respectively. The measurement of the temperature was taken by following the laser spot, so that the pyrometer was always coincident with the melt pool. Once the experimental tests were finished, the different tracks were cut in a wet abrasive cut-off machine with a corundum cut-off wheel bonded with rubber and then etched with Kalling’s reagent 2. The cross sections were analyzed by means of a confocal microscope Leica DCM 3D (Leica Microsystems GmbH, Wetzlar, Germany). For the cross section characterization, four main parameters were measured (See Figure 2), including height, width, depth of dilution, and wet angle [18,19].

## 3. Results

### 3.1. Analysis of the Melt Pool Temperature and Cooling Time

A preliminary analysis of the temperature indicates that measurements taken before the first 500 to 800 ms are very unstable. This is due to the different phenomena occurred during the rapid rise of the temperature that mislead the pyrometer sensor. For this reason, the time while the laser was radiating the substrate was set to one second. The results show the same order of magnitude of temperature for the three studied gases.

**Figure 3:**
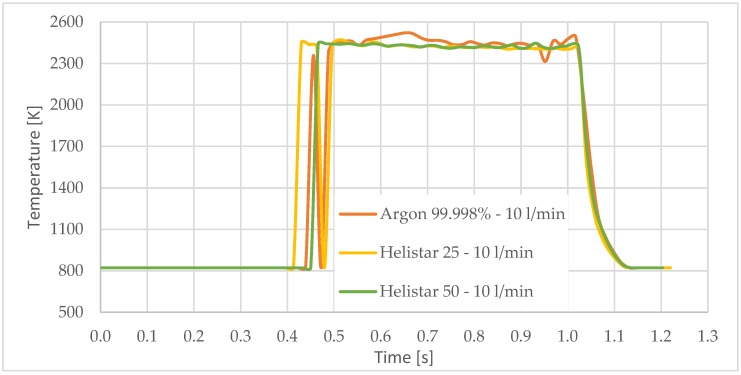
Temperature [K] vs. time [s].

Figure 3 shows the measurement results. The pyrometer has a minimum temperature range of 823 K on display, and a maximum of 2773 K, and it was calibrated before the experiments.

In all cases, temperature reaches a mean value of approximately 2420 K at the time of 1 s, and it takes approximately 130 ms (131 ms for Argon 99.998%, 130 ms for Helistar 25, and 138 ms for Helistar 50) from that point to reach a temperature below 823 K approximately. These results imply that, when working with low laser power (such as 250 W), there is no significant difference between the mixtures that were studied.

### 3.2. Analysis of the Laser Metal Deposition Experiments

Along these tests, the influence of different gas mixtures on the LMD process was analyzed for three different laser powers: 400, 600, and 800 W. The following figures illustrate the experimental tests and the resulting tracks for the different gas mixture. The morphology of the clads is represented and measured, followed by a table with average values of each of the three repetitions of the experimental tests.

#### 3.2.1. Experimental Tests with 400 W

Table 5 shows that, for the same energy density value, width, dilution depth and height of the track decrease as the concentration of helium grows. The results show very similar height, width, and dilution in all cases. Wet angle presents also differences between the tests, but the main variation is for the Helistar 50.

Regarding the wet angles, the optimal values should not exceed 65°. In this case, the parameter combination of 400 W laser power and selected powder mass flow is not adequate to obtain optimum wet angles.

Due to possible variations in the measurements of the clads, the experimental tests were repeated three times. Average values of the measurements are presented in Table 6 with average absolute deviation around the mean (MAD) value in brackets.

#### 3.2.2. Experimental Tests at 600 W

Continuing with the same methodology, tests with a laser power of 600 W are presented in Table 7. There are significant increments in width due to the higher power, but not in height, which mainly depend on the powder mass flow. For this laser power, the width of the different clads hardly variates among gas mixtures as well as the depth of material dilution. However, the variation of the height as a result of the use of different gases is more significant, and, again, a greater variation is observed for the highest helium concentration tests (Helistar 50).

Table 7 shows also a reduction of wet angles of the clads due to the higher laser power. However, significant differences of wet angle are observed for clads made with each gas mixture. The higher the helium concentration, the smaller the wet angle of the clad.

Again, a repetition of the tests was made in order to consider the possible variations. The results with average values are presented in Table 8. As it can be observed, height, width, and dilution depth values are practically constant, regardless of the gas composition. However, the wet angles vary considerably for the different gas mixtures.

#### 3.2.3. Experimental Tests at 800 W

Tests results for 800 W laser power are shown in Table 9. Because of power increment, the width and dilution are higher than those obtained in the 400 W and 600 W tests.

Similarly to the previous experiments, the highest variation between the clads is in height, whereas the dilution depth and width remain almost invariable.

Because of a higher laser power, the wet angle is smaller than previous tests with lower laser power. In addition, the wet angle changes for different gas compositions, as it can be observed in Table 9. The wet angle reduction is the most significant variation when using different gases, while the rest of measurements present similar values.

The experimental tests once again are repeated to obtain more information and to analyze the possible variation on the geometry of the clad. Table 10 shows these results as mean values of the measurements. As it was stated before, the more significant effect of the different gases use is the wet angle variation, and this variation is more considerable for higher helium concentrations.

#### 3.2.4. Temperature Analysis

In addition to the geometry analysis, the temperature of the process was registered via pyrometry and Table 11 shows the outputs of these measurements.

In all cases, the utilization of helium in the process results in higher temperature. When pure argon was used, mean temperatures of 1938 K, 1991 K, and 2121 K were reached for laser powers of 400 W, 600 W, and 800 W, respectively. Meanwhile, when Helistar 25 (Ar 75% and He 25%) and Helistar 50 (Ar 50% and He 50%) was employed, temperatures with average values of 2093 K and 2097 K where measured, respectively, at 400 W. However, increasing the amount of helium in the mixture did not result in a higher temperature.

## 4. Discussion

As it has been observed in the previous tests, the helium content of the gas affects the geometry of the deposited clad. The comparison between the tests that were realized with argon and those with Helistar 50, shows height value differences of 70 µm, 90 µm, and 140 µm for laser power values of 400 W, 600 W, and 800 W, respectively. These height variations are slightly lower when Helistar 25 is used. With regard to width and depth values, the differences do not exceed 30 µm for dilution depth and 50 µm for width, and no clear tendency is appreciated. Moreover, the wet angle seems to have a correlation with the helium concentration of the gas, changing the shape of the clad geometry by smoothing the slope. The wet angle decreases when the helium proportion is increased and variation values up to 21 degrees are registered.

The melt pool temperature variation is other factor, which is strongly influenced by the gas mixture. With argon, variations of almost 200 K were registered from a 400 W to 800 W laser power. However, the same differences of laser power do not show significant variations of the temperature when gases with presence of helium are used, merely of 20 K approximately.

As Andreas Patschger and Rolf Wester et al. state [6,7], the ionization energy of the gas is important due to the formation of plasma in the laser beam way. In this case, argon is more susceptible of forming ionized gas because of its lower ionization energy value, which is near to the 64% of the helium’s one. In addition, the heat conductivity of these two gases are very different, being helium’s (0.151 W·m^−1^·K^−1^) near 40 times higher than argon’s thermal conductivity (0.018 W·m^−1^·K^−1^).

Plasma works as an isolation for the laser beam and higher heat conductivities allow for the heat that is radiated to be fed back to the process. The variation of the temperature for the different gases can be explained with these two phenomena. For a high energy density process, the use of argon contributes to plasma formation, and thus, the isolation of part of the energy provided by the laser. On the other hand, helium is able to work with higher energy densities without promoting plasma formation and contributing to feeding back heat to the process due to its greater thermal conductivity.

## 5. Conclusions

The present work studies the influence of the gas composition on the LMD process. Three different gas compositions have been tested: Ar 99.99%, Ar 75%-He 25%, and Ar 50%-He 50%. Up to 150 microns, differences in height are observed between Ar 99.99% and Ar 50%-He 50% concentration gases. The higher the helium presence in the mixture, the smaller the height of the clad. The rest of the geometry characteristics remain virtually stable with a variation of less than 60 microns for extreme cases.

The most significant variation on the shape of clads is the wet angle. This parameter variates within a wide range with the helium concentration, decreasing its value while the presence of this gas goes in augment. Variations of 10 to 20 degrees are observed between the use of pure argon and a mixture with 50% of helium. In addition, the temperature of the melt pool is also influenced by the presence of helium when it is combined with high energy densities.

Conclusions of the present research work can be summarized in the following way:

1. Helium and argon process gases have different effects on LMD. Its influence is not negligible and must be taken into account.
2. The increment of helium concentration in the gases of the LMD processes based on argon will have three effects. The first one is a slight reduction of the height of the clads. Second, an increase of the temperature of the melt pool. Last, smaller wet angles are obtained for higher helium concentrations.
3. However, some variations can be neglected due to its small values, like width and dilution depth, since helium concentration seems to have no special influence on these parameters.

## Figures and Tables

**Table 1:** Chemical composition (wt %) of the Inconel 718 substrate [12].

Ni	Cr	Cb + Ta (Nb + Ta)	Cb (Nb)	Mo	Ti	Al	Co	Mn
52.50	18.40	5.08	5.08	3.04	1.03	0.54	0.33	0.24
**Si**	**C**	**Cu**	**B**	**Ta**	**P**	**S**	**Fe**	
0.11	0.052	0.05	0.005	<0.05	0.006	<0.002	BAL	

**Table 2:** Chemical composition (wt %) of the MetcoClad 718 powder [13].

Cr	Mo	Nb	Fe	Ti	Si	Mn	C	B	Ni
19.00	3.00	5.00	18.00	1.00	0.20	0.08	0.05	0.005	BAL

**Table 3:** Particle size, which follows a Rosin-Rammler distribution.

Minimum Size (µm)	Mean Size (µm)	Maximum Size (µm)	Spread Parameter	Number of Diameters
50	90	135	4.2	10

**Table 4:** Laser Metal Deposition (LMD) experiments process parameters.

Reference	Power (W)	Feed Rate (mm·min^−1^)	Spot Diameter (mm)	Powder Mass Flow (g·min^−1^)	Shielding Gas Flow (L·min^−1^)	Dragging Gas Flow (L·min^−1^)
[1]	400	500	1.6	8.0	12.0	4.5
[2]	600	500	1.6	8.0	12.0	4.5
[3]	800	500	1.6	8.0	12.0	4.5

**Table 5:** Measurements for different gases at 400 W.

Argon 99.998%	Ar 75% He 25%	Ar 50% He 50%
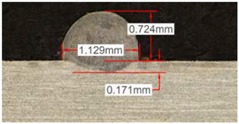	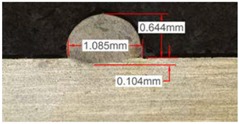	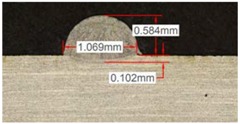
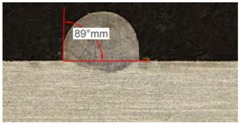	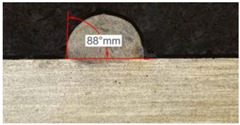	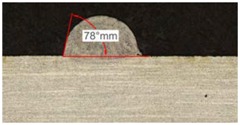

**Table 6:** Summary of results for experimental tests at 400 W. (absolute deviation around the mean (MAD) in brackets).

Gas Mixture	Height (mm)	Width (mm)	Depth (mm)	Wet Angle (°)
Argon 99.998%	0.69 (0.040)	1.15 (0.018)	0.16 (0.013)	89 (1.0)
Ar 75% He 25%	0.63 (0.020)	1.10 (0.014)	0.13 (0.023)	88 (1.5)
Ar 50% He 50%	0.60 (0.029)	1.06 (0.009)	0.10 (0.009)	78 (1.0)

**Table 7:** Measurements for different gases at 600 W.

Argon 99.998%	Ar 75% He 25%	Ar 50% He 50%
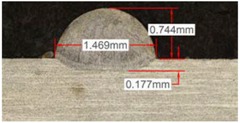	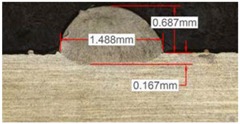	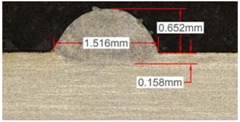
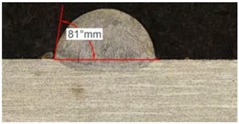	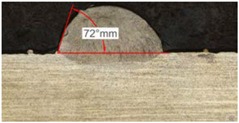	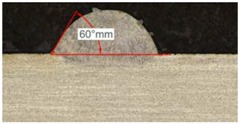

**Table 8:** Summary of results for experimental tests at 600 W.

Gas Mixture	Height (mm)	Width (mm)	Depth (mm)	Wet Angle (°)
Argon 99.998%	0.75 (0.009)	1.48 (0.015)	0.18 (0.004)	81 (1.2)
Ar 75% He 25%	0.70 (0.016)	1.49 (0.009)	0.17 (0.008)	73 (1.0)
Ar 50% He 50%	0.66 (0.009)	1.51 (0.010)	0.15 (0.009)	60 (0.8)

**Table 9:** Measurements for different gases at 800 W.

Argon 99.998%	Ar 75% He 25%	Ar 50% He 50%
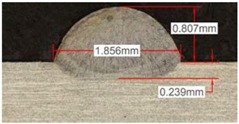	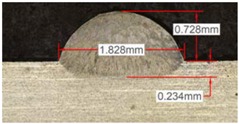	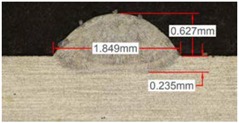
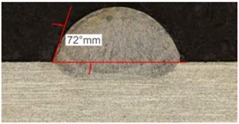	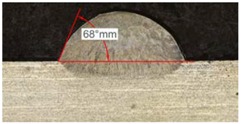	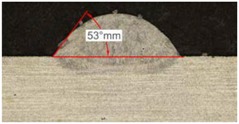

**Table 10:** Summary of results for experimental tests at 800 W.

Gas Mixture	Height (mm)	Width (mm)	Depth (mm)	Wet Angle (°)
Argon 99.998%	0.79 (0.014)	1.87 (0.013)	0.25 (0.010)	73 (1.0)
Ar 75% He 25%	0.73 (0.009)	1.82 (0.015)	0.26 (0.008)	67 (1.2)
Ar 50% He 50%	0.65 (0.020)	1.84 (0.010)	0.23 (0.006)	53 (0.8)

**Table 11:** Summary of results for experimental tests at 400 W, 600 W, and 800 W.

Argon 99.998%	Helistar 25	Helistar 50